# The Multifaceted Impact of Karrikin Signaling in Plants

**DOI:** 10.3390/ijms26062775

**Published:** 2025-03-19

**Authors:** Qilin Deng, Hongyang Wang, Yanhong Qiu, Dexin Wang, Yang Xia, Yumeng Zhang, Manying Pei, Yinling Zhao, Xiulan Xu, Haijun Zhang

**Affiliations:** 1 State Key Laboratory of Vegetable Biobreeding, Beijing Vegetable Research Center, Beijing Academy of Agriculture and Forestry Science, Beijing 100097, China; dengqilin0905@163.com (Q.D.); wanghongyang@nercv.org (H.W.); qiuyanhong@nercv.org (Y.Q.); wangdexin@nercv.org (D.W.); xiayang@nercv.org (Y.X.); zymeng0615@163.com (Y.Z.); peimanying3331745@163.com (M.P.); 18232151732@163.com (Y.Z.); 2 College of Horticulture, China Agricultural University, Beijing 100193, China; 3 National Engineering Research Center for Vegetables, Beijing Vegetable Research Center, Beijing Academy of Agriculture and Forestry Science, Beijing 100097, China

**Keywords:** biological function, karrikins (KARs), KARRIKIN INSENSITIVE2 (KAI2), plant growth, stress tolerance, signal transduction

## Abstract

Karrikins (KARs), produced during wildfires, are bioactive compounds that stimulate seed germination in fire-prone ecosystems and influence broader plant–environment interactions. These compounds act through the α/β hydrolase receptor KARRIKIN INSENSITIVE2 (KAI2), which perceives KARs as analogs of the hypothesized phytohormone KAI2 ligand (KL). KAR signaling shares molecular parallels with strigolactones (SLs), another class of butenolide plant hormones, and regulates diverse processes such as seedling development, root architecture, photomorphogenesis, and stress responses. Despite its multifaceted roles, the mechanistic basis of KAR-mediated regulation remains poorly understood. This review synthesizes insights into KAR signaling mechanisms, emphasizing recent advances in signal transduction pathways and functional studies. It also addresses key unresolved questions, including the identity of endogenous KL and the crosstalk between KARs and other hormonal networks. By elucidating these mechanisms, KAR-based strategies hold promises for enhancing crop resilience and sustainability, offering novel avenues for agricultural innovation in changing environments.

## 1. Introduction

Fire is an important evolutionary force promoting regeneration in many plant communities [1,2]. Karrikins (KARs) are a class of butenolide compounds produced in wildfire smoke and play a crucial role in fire-dependent plant community recovery [3,4]. The first KAR, 3-methyl-2*H*-furo[2,3-*c*]pyran-2-one, was identified by using nuclear magnetic resonance in conjunction with chromatographic analysis in 2004 and named KAR_1_ [5]. Further studies identified five additional KARs, namely KAR_2–6_ [6,7]. KARs are known to promote seed germination, but the specific mechanism remains unclear. A study demonstrated that KARs can induce seed germination and seedling growth in *Arabidopsis thaliana*—a model plant that typically does not experience fire [8]. This breakthrough led to the realization that KARs could influence fire-adapted species and prompted research in KAR signaling.

KARRIKIN INSENSITIVE 2 (KAI2), encoding an α/β hydrolase protein, is required for KAR-mediated seed germination in Arabidopsis [9]. In vitro binding assays and protein–ligand co-crystallization suggested that KAI2 can perceive KARs [10,11]. KAR signaling via KAI2 requires an F-box protein MORE AXILLIARY GROWTH 2 (MAX2) from the Skp, Cullin, F-box (SCF)-type E3 ubiquitin ligase complex [9]. Upon activation, KAI2 with SCF^MAX2^ triggers the ubiquitination and degradation of KAR-specific SUPPRESSOR OF MAX2-LIKE (SMXL) proteins, SMAX1 and SMXL2 [12,13,14], driving seed germination via the KAI2 signaling cascade. Beyond germination, KAR signaling influences significant processes like hypocotyl elongation, root hair development, and responses to abiotic stress [14,15], highlighting KAR signaling as a versatile mechanism for plant adaptation to varying environments and its potential to improve plant growth and stress tolerance. However, these mechanisms are still poorly understood, and further research is needed to explore their full potential.

Despite these advances, critical questions about the KAI2 ligand (KL), the proposed endogenous ligand for KAI2, remain unresolved [13,14]. Understanding the structure and function of KL is essential for fully understanding the KAR signaling pathway. Additionally, while KARs promote seed germination, their interactions with other plant hormones and signaling pathways, including the role of SMXL proteins as potential downstream regulators, are not yet fully understood [15].

In this review, we aim to provide a comprehensive overview of the current knowledge on KAR signaling, focusing on the structural characteristics of the signaling pathway components and the mechanisms by which KARs are perceived and transduced by KAI2. We also discuss the biological functions regulated by KAR signaling and highlight the gaps in our understanding that need to be addressed. Specifically, we emphasize the need for further research to elucidate the perception of KAR signals by KAI2, identify downstream target genes of SMXL proteins, and explore the functional diversity of SMXLs across different plant species. Ultimately, these efforts will contribute to a deeper understanding of KAR signaling and its potential applications in agriculture and plant biology.

## 2. Structure of KARs

**Figure 1:**
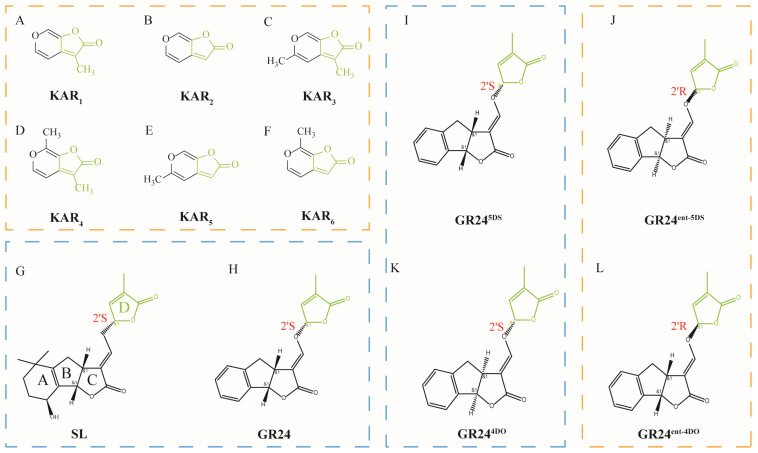
Schematic structural diagram of KARs and SL analogs. (**A**–**F**) Structure of KAR_1_–KAR_6_. All KARs are composed of two rings: a six-membered pyran ring and a five-membered butenolide ring. The number and position of the methyl groups connected to the ring distinguish the six KARs. (**G**,**H**) Structure of natural SL and synthetic GR24. (**I**–**L**) Structures of the commonly used synthetic rac-GR24. GR24^5DS^ (**I**) and GR24^4DO^ (**K**) function similarly to SL. GR24*^ent^*^-5DS^ (**J**) and GR24*^ent^*^-4DO^ (**L**) function similarly to KARs. Green structures are the variations that distinguish them.

KAR_1_~KAR_6_ comprise a six-membered pyran ring fused with a five-membered butenolide ring [16], differing only in their methyl substitutions [6] (Figure 1A–F). Strigolactones (SLs) share a core structure of four rings, with an ABC-ring system linked to a butanolide D-ring with a 2’*R* configuration [17]. The conserved C- and D-rings are important for their activity (Figure 1G). The butenolide ring of KARs is closely related to the lactone D-ring of SL [16] (Figure 1A,G).

Despite the structural similarity, KARs and SLs exhibit distinct biological activities, which can be attributed to differences in their stereochemistry and functional groups. For instance, synthetic analogs of SLs, such as GR24, have been shown to act as KARs depending on their stereochemistry [18,19]. Research on four synthetic SL analogs revealed that GR24^4DO^ (same stereochemistry as 4-deoxyorobanchol) and GR24^5DS^ (same stereochemistry as 5-deoxystrigol) functioned similarly to SLs, whereas GR24*^ent^*^-4DO^ (same stereochemistry as *ent*-4-deoxyorobanchol) and GR24*^ent^*^-5DS^ (same stereochemistry as *ent*-5-deoxystrigol) acted as KARs [18]. Moreover, while *ent*-5-deoxystrigol (5DS) and 4-deoxyorobanchol (4DO) are natural SLs, their synthetic enantiomers, *ent*-5-deoxystrigol (*ent*-5DS) and *ent*-4-deoxyorobanchol (*ent*-4DO), also act as KARs [20]. The deoxysls 4DO and 5DS (including their enantiomers *ent*-4DO and *ent*-5DS), isolated from rice and tobacco root exudates, have the same SL skeleton [21]. The distinguishing factor between whether these compounds respond to SLs or KARs is the D-ring configuration, with the responsive SLs having a 2’R configuration and the responsive KARs having a 2’S butenolide ring [22,23,24,25]. In addition, recent studies have shown that natural KARs are unstable and do not undergo chemical modification. KARs may not activate KAI2 directly. However, GR24*^ent^*^-5DS^ can activate KAI2, and its stimulation to plants is stronger than KARs [12,26,27,28,29]. This makes GR24*^ent^*^-5DS^ a widely used substance in studying KAR signal transduction, further accelerating the research process on KAR. Understanding these structural details could facilitate the development of more potent and specific KAR analogues for agricultural applications.

## 3. Signal Transduction of KARs

**Figure 2:**
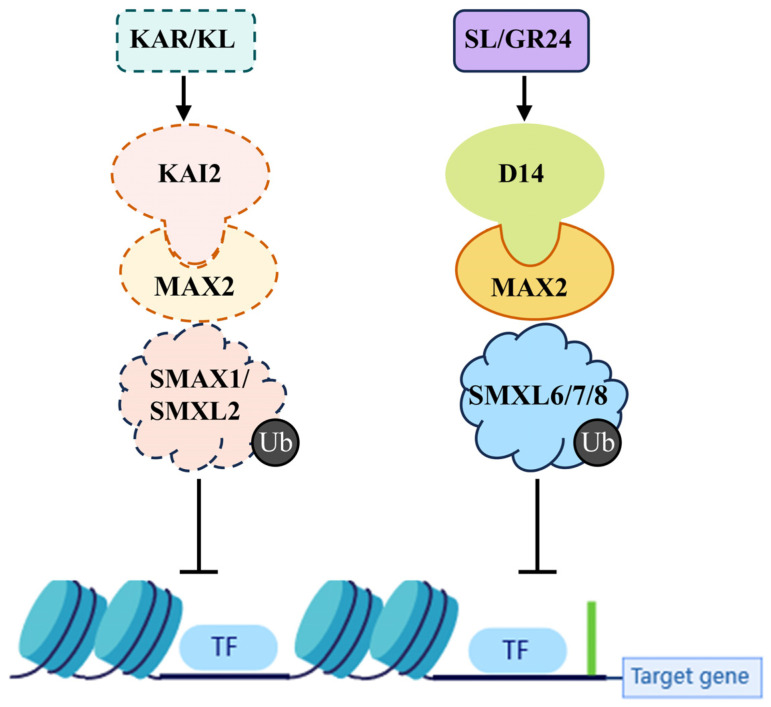
Simplified model of SL and hypothetical KAR Signaling. SL binds to the D14 receptor. This interaction initiates the formation of the D14-SCF^MAX2^-SMXL6/7/8 complex for ubiquitination and subsequent degradation. KAR or assumed KLs are detected by the KAI2 receptor. Ligand–receptor interaction triggers the KAI2-SCF^MAX2^-SMXL1/SMXL2 complex, which induces SMXL1/SMXL2 ubiquitination and degradation. Consequently, the inhibition of unknown transcription factors is relieved, activating downstream target expression. Both SL and hypothetical KAR signaling pathways share the SCF complex protein MAX2.

The KAR signal transduction pathway resembles that of SLs and can be categorized into three levels. First, the receptor protein KAI2 detects KARs/GR24, subsequently forming an SCF complex with F-box protein MAX2 (Figure 2). This complex triggers the ubiquitination and degradation of the transcription suppressor SMAX1/SMXL2, alleviating the inhibition of downstream gene expression and regulating plant growth and development [12,13,30].

### 3.1. The KAR Receptor KAI2 Protein

#### 3.1.1. The Structure of KAI2 Protein

**Figure 3:**
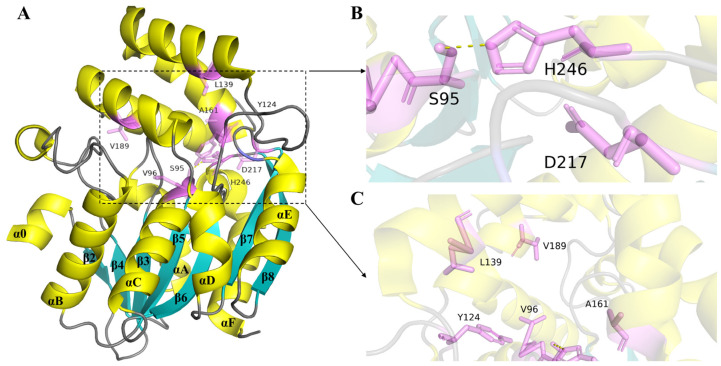
Schematic diagram of the three-dimensional structure of the AtKAI2 protein. (**A**) The figure marks the secondary structure of the KAI2 protein, αo–αF, and β2–β8. (**B**) Catalytic triad structure (S95-H246-D217). (**C**) The five amino acid positions of the KAI2 protein: V96, Y124, L139, A161, and V189. The yellow part is the α helical structure, and the blue part is the β folded part. The pink stick-like structure represents amino acids.

Structural studies of KAR receptors have provided critical insights into the molecular mechanisms of KAR perception [31,32]. KAI2 was initially discovered through photomorphogenesis mutant screening and subsequently named HYPOSENSITIVE TO LIGHT (HTL) [31]. Both the KAI2 protein and the Dwarf 14 (D14) protein belong to the α/β hydrolase superfamily and possess a conserved catalytic triad structure (Ser–His–Asp) (Figure 3B). These proteins serve as atypical plant hormone receptors, exhibiting both hydrolase and receptor functions [32]. The overall structure of AtKAI2 has folded β-strands (β2–β8) surrounded by five helices (αo, αB–αE) on one side and two helices (αA and αF) on the other (Figure 3A). The top region of the protein is a double V-shape formed by the four helices (αA–αB), and at the bottom of the double V-shape is the substrate-binding orifice [11]. The α-helix and β-folded core are connected to a cap composed of two pairs of V-shaped α-helices. The conserved catalytic triplet is situated between these two domains, forming the foundation of a hydrophobic substrate-binding pocket [33].

Analyzing the molecular basis of preference facilitates the prospective development of ligands that can elicit highly responsive effects. Structural homology modeling had pinpointed specific residues near KAI2’s binding pockets that may influence ligand selectivity. Variations in KAI2 proteins from different species are notably found at amino acid positions 96, 124, 139, 161, and 189 (Figure 3C) [27,34,35,36], with sites 96 and 189 linked to functional differences [34]. These findings underscore the evolutionary conservation of KAI2 and its evolving ligand preferences for diverse KARs. Recent studies reveal that KAI2 receptors are not limited to detecting KARs and SLs but also respond to sesquiterpene lactone compounds [37,38]. For instance, the petunia receptor PhKAI2ia specifically senses (−)-germacrene D, a sesquiterpene emitted by PhTPS1 in the flower, initiating a KAI2-mediated signaling pathway crucial for plant fitness [38]. This broadens our understanding of KAI2’s role in perceiving a spectrum of volatile compounds, underscoring its potential for diverse applications. In summary, the structure of KARs and their receptors provides a foundation for understanding the molecular mechanisms of KAR signaling. The structural similarity to SLs and the involvement of conserved signaling components, such as MAX2, underscore the evolutionary and functional connections between these two classes of plant hormones. Future research should focus on elucidating the structural determinants of KAR activity and exploring the functional diversity of KAI2 homologs to fully harness the potential of KAR signaling in plant biology.

#### 3.1.2. Functional Significance of KAI2 Hydrolysis

The hydrolytic activity of KAI2 and D14 towards diverse butenolide substrates is attributed to their conserved catalytic triad comprising Ser, His, and Asp residues [39,40]. While the signaling function’s biological relevance remains unclear, receptors with mutated catalytic residues cannot initiate signal transduction, suggesting ligand hydrolysis is essential [39,41].

Based on crystal structures of D14 homologs in Arabidopsis and rice, we hypothesize that D14 facilitates SL molecule hydrolysis, resulting in D-ring separation from the ABC scaffold and subsequent vacating from the catalytic site [41]. The open D-ring covalently binds to the catalyzed Ser residue and subsequently transfers to the catalyzed His residue, resulting in the formation of “covalently linked intermediate molecules” (CLIMs) [33,42]. These intermediates may induce receptor conformational changes stabilized by partners like MAX2, highlighting the role of proteolysis and receptor–protein interactions in KAR signal transduction [43,44].

KAI2’s hydrolysis mechanism is hypothesized to resemble D14’s, though direct evidence has been limited. A recent X-ray crystallographic study of pea PsKAI2B has validated this speculation, revealing 5-hydroxy-3-methylbutenolide bound to catalytic S [26]. By mass spectrometry analysis, this compound may represent the first reaction intermediate after nucleophilic attack on the butenolide carbonyl. The hydrolysis reaction leads to covalent modification of the catalytic triad, and the cleaved butenolide ring can open to bridge Ser and His residues to form a CLIM or bind to Ser or His residues individually [26]. This model is further supported by molecular dynamics and quantum mechanical free energy simulations [45,46]. In summary, these studies suggest that KAI2 and D14 share a similar signaling mechanism involving ligand hydrolysis and conformational changes.

#### 3.1.3. KL, the Endogenous Ligand for KAI2

KARs are widely recognized as the primary ligands for KAI2 receptors [11,47,48]. However, their efficacy as ligands is limited in species like Arabidopsis, which are unadapted to fire, making KARs less effective for KAI2 signaling [49,50,51]. Furthermore, recent research showed that unmodified KAR does not activate KAI2 and initiate signal transduction [12,52]. This has led to a new hypothesis: KAI2 primarily recognizes other KLs, not KARs. The specific identity of the compound is still a mystery, yet it is speculated to be a hydrophobic butenolide compound [13,53]. In Arabidopsis, (−)-desmethyl GR24, a synthetic SL analog, better mimics KL effects than its methylated version [54], suggesting a demethylated butenolide origin for KL [13,54]. Additionally, the KARRIKIN UP-REGULATED F-BOX 1 (KUF1) protein has been identified as a negative regulator of KAI2 signaling, implying that KL may be involved in regulating the stability and activity of KUF1 [55]. Identifying KL is challenging due to its likely low concentration in plant tissues, structural diversity among candidates, and the complexity of its conserved biosynthetic pathway [13,55]. Understanding KL is critical for uncovering KAR signaling mechanisms and their roles in plant development and stress responses. Future research should focus on elucidating the structure and biosynthetic pathways of KL. Advances in metabolomics and genetic engineering may aid this effort while studying KAI2 homologs across species could reveal species-specific KLs and their ecological roles.

### 3.2. The Coreceptor MAX2

The MAX2 protein, derived from KAR-insensitive mutants, serves as a pivotal signal transducer that integrates the SL and KAR signaling pathways [56]. It is an F-box protein essential for the ubiquitination process in hormonal signaling systems [57,58]. The C-terminal of the F-box protein, characterized by features such as leucine zippers and zinc finger motifs, facilitates its interaction with substrates [59]. MAX2 is demonstrated to interact with KAI2 and D14 in a KAR-dependent manner [38,49,60,61].

In rice, the MAX2 homolog D3 binds to D14 through its leucine-rich repeat (LRR) region, capturing covalent GR24-hydrolyzed ligands in binding pockets [33]. The C-terminal helix (CTH) of D3 exhibits conformational flexibility, switching between a closed, helical structure and an open, crimped state [42]. The CTH’s conformational state determines SMXL substrate recruitment for proteasomal degradation during substrate signaling, influencing KAI2 recruitment and SMAX1 targeting in the KL signal [62,63]. These conformational dynamics underscore the critical role of the D3/MAX2–CTH model in KAR signaling.

Understanding the mechanisms by which MAX2 functions in KAR signaling is crucial for elucidating the regulatory networks that govern plant growth and development. Future research should focus on elucidating the structural basis of MAX2’s interactions with KAI2, as well as its role in the degradation of SMXL proteins.

### 3.3. The Repressor SMXL Proteins

**Figure 4:**
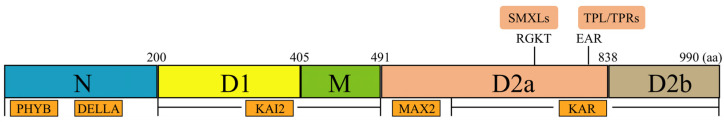
SMAX1 protein and its functional domain. The SMAX1 protein consists of five domains: double Clp-N motif (N), putative ATPase domain 1 (D1), middle region (M), putative ATPase domain 2a (D2a), and D2b in the N-terminal to C-terminal direction. SMAX1 interacts with signaling molecules such as phytochrome B (phyB) and DELLA in the N domain. The mutual binding of SMAX1 and KAI2 receptors depends on the D1 and M domains. The front end of the D2a domain is responsible for binding to the MAX2 protein and then senses KAR signals together with the D2b domain. The D2a domain also contains the RGKT motif and the conserved EAR motif. The RGKT motif is responsible for the MAX2-dependent degradation of the SMAX1 protein. The EAR motif is involved in the interaction of SMAX1 with TOPLESS (TPL) and TPL-RELATED (TPR).

The SMXL family, first identified through SMAX1’s role in suppressing seed germination and early seedling development in *max2* mutants [64], consists of eight members in Arabidopsis, categorized into four subgroups: SMAX1/SMXL2, SMXL3, SMXL4/5, and SMXL6/7/8, each with unique structural and functional traits [65,66]. SMXL proteins share domain organization and certain key motifs with the casein-breaking peptidase B (ClpB) ATPase family [67], including conserved N-terminal double Clp-N motifs and nucleotide-binding domains (NBDs) (NBD1 and NBD2, D1 and D2) containing Walker A and B motifs, separated by M domains [65,66,68]. The feature that distinguishes SMXL proteins from other Clp ATPases is the ethylene response factor-associated amphiphilic repression (EAR) motif between the two NTPase subdomains in D2, as well as an extended M domain [67]. In KAR signaling, SMXLs are degraded by the RGKT motif, and SMAX1 was able to inhibit gene expression in an EAR-dependent manner [52,69]. SMAX1 is a key regulator in the KAR signaling pathway, with specific domains playing essential roles (Figure 4). The D2 domain guides the nuclear localization, while the D1 and M domains facilitate interaction with KAI2. The RGKT and EAR motifs function similarly to those in SMXL6/7/8 [52,70]. The role of SMAX1 as a transcription factor has been debated. A recent study found for the first time that SMAX1 binds to its promoter for self-inhibition and the promoters of *Gibberellin 3-oxidase 1* (*GA3ox1*) and *Gibberellin 3-oxidase 2* (*GA3ox2*) affect gibberellic acid (GA) synthesis. The main binding motif is TTGTTAT/ATAACAA [71]. Under red light, SMAX1/SMXL2 regulate PIF4/PIF5 levels via a non-transcriptional process, affecting cotyledon angle development, independent of the EAR motif [72]. This challenges the conventional view that the SMXL family modulates downstream genes solely through transcriptional regulation, suggesting broader functional roles.

D14-MAX2-SMXL6/7/8 and KAI2-MAX2-SMAX1/SMXL2 are the typical signal transduction pathways that participate in plant branching, hypocotyl elongation, seed germination, etc. [73,74,75]. However, recent studies have found that the physiological functional regulation of SMXL family proteins is not independent, but there are cross-effects. For example, in the regulation of hypocotyls, SMXL2 responds not only to the KAR signaling pathway but also to the SL signaling pathway [12,30]. Further study has also found that SMAX1 is also required for D14-dependent suppression of hypocotyl elongation [76]. Currently, our comprehension and examination of SMXL-regulated physiological processes are limited, and further research to clarify the molecular mechanisms is needed [77].

## 4. Functions of KARs

Genetic research on model organisms such as Arabidopsis and rice has demonstrated the indispensability of KARs in a variety of biological processes, encompassing seed germination, seedling growth, developmental regulation, stress tolerance, pigment biosynthesis, root morphology, and the establishment of symbiotic relationships with arbuscular mycorrhizal fungi (AM) [38,78,79,80,81,82,83,84,85,86]. These processes are regulated through a complex network of hormonal interactions, not solely by KARs [87,88].

### 4.1. Seed Germination

Notably, over 1200 plant species have been identified as displaying positive germination responses to smoke or smoke water, including many crops [89]. KAR_1_, even at very low concentrations (around 10^−9^ M), can induce germination, highlighting its potential as a seed primer [90]. KARs stimulate germination by interacting with ABA and GA [11,71,91]. *CYP707A* genes were upregulated upon KAR treatment in both parasitic plants and Arabidopsis [92]. AtHTL/KAI2 negatively regulates SMAX1 in *Stiga* and Arabidopsis, and ligand-dependent SMAX1 inactivation bypasses GA to germination [91]. ShHTL7 and its *smax1-2* mutants are ABA-insensitive, while the *bri1-6* mutant, which reduces BR sensitivity, may hinder ShHTL7’s ability to overcome dormancy by increasing *ABI3* expression [93]. A synthetic desmethyl-type germinone (dMGer) specifically targets KAI2 and induces GA-independent germination in Arabidopsis [94]. KAR signaling under low light leads to the degradation of the SMAX1 protein, consequently relieving its inhibition of GA3ox1 and GA3ox2, thereby promoting seed germination [71]. KARs also regulate seed germination through light responses [95]. The interaction between KAR_1_ and red/far red light (R/FR) signaling is also hormone-related [96,97]. Light promotes GA biosynthesis by activating GA3ox genes via the phytochrome system and reduces ABA and IAA levels by inhibiting the *ABA2* and *YUCCA* genes, respectively [98]. Interestingly, KAR_1_ regulates some light-induced genes, such as *GA3oxs* and *YUCCA* [71,98]. PHYTOCHROME-ASSOCIATED PROTEIN PHOSPHATASE5 (PAPP5) may modulate KAI2-dependent seed germination by dephosphorylating MAX2 [99]. In addition, KAR_1_ can also interact with NO to induce seed germination by stimulating the production of ethylene biosynthetase (ACC), oxidase (ACO), and ACC synthetase (ACS) [100,101]. The ability of KARs to enhance seed germination has significant practical applications in agriculture. KARs can be used to improve seed performance in crops, particularly in species that exhibit high dormancy or poor germination under stress conditions.

### 4.2. Hypocotyl Growth and Seedling Photomorphogenesis

Photomorphogenesis and hypocotyl elongation are essential for plant development, with the KAR signaling pathway involved in their regulation [84,102]. SMAX1 and SMXL2 regulate hypocotyl growth and the expression of KAR/SL transcriptional markers *KUF1* and *DWARF14-LIKE2 (DLK2)* redundantly in Arabidopsis [102]. Double mutants of *smax1* and *smxl2* were insensitive to KARs and SLs in hypocotyl growth [12,102]. A mutant in Arabidopsis named *pleiotropic long hypocotyl2 (ply2)*, a missense allele of KAI2, was found to affect photomorphogenic seedling development [103]. Overlapping response genes were identified between the KAR signaling and light signaling pathways [103]. KAI2-KL signaling functions downstream of *CONSTITUTIVELY PHOTOMORPHOGENIC1* (*COP1*), a key negative regulator of photomorphogenesis, and is largely independent of *LONG HYPOCOTYL IN FAR-RED* (*HFR1*). *KAI2* expression is induced by *ELONGATED HYPOCOTYL 5 (HY5)*, and exogenous application of KAR inhibits hypocotyl elongation of *cop1*-6 in darkness [103]. The HY5-BBX transcription module is induced by KAR signaling, and RNA sequencing of *hy5* showed the promotion of hypocotyl elongation by SMAX1 and SMXL2 was dependent on BBX20 and BBX21 [79]. In addition, SMAX1 interacts with the light signal receptor Phytochrome B (phyB) to relieve the PHYTOCHROME-INTERACTING FACTOR 4 (PIF4) activity, promoting thermal hypocotyl regeneration and enhancing thermal sensitivity in Arabidopsis [104].

KAI2 and MAX2 depend on SMAX1 to limit the hypocotyl growth associated with shade avoidance in Arabidopsis, providing insights for improving crop yields under low-light conditions [105]. Interestingly, in monocots such as rice, KAR inhibits mesocotyl elongation which plays an analogous role to the hypocotyl in a KAI2-dependent manner without light [82], unlike the light dependency in Arabidopsis, suggesting KAR-KL signaling can operate independently of light.

KAI2 also regulates seedling morphogenesis through the auxin transport system [84]. Reduction in PIN3, PIN4, and PIN7 abundance in the hypocotyl and an increase in PIN1, PIN3, and PIN7 abundance at the root meristem are thought to stall hypocotyl elongation [84]. Overexpressing *SMAX1* in Arabidopsis affected the expression of many auxin homeostasis genes, especially under the simulated shade process, increasing the hypocotyl shade response [105]. During the dark-to-light transition, SMAX1-mediated degradation of DELLA is elevated in seedling establishment, attenuating DELLA action on hypocotyl growth [106]. It suggested that SMAX1 can integrate both the light and KAR signals into the GA pathway during seedling establishment.

### 4.3. Regulation of Root Development

The KAR signaling pathway impacts four key aspects of root system architecture: root hair growth, root skewing, lateral root proliferation, and nodule formation [81,85,107,108,109]. KAR signal transduction promotes root hair elongation by affecting IAA and ethylene [81,85]. KAI2 can control the accumulation of AUXIN TRANSPORTER PROTEIN1 (AUX1) and PIN-FORMED2 (PIN2), inhibit the expression of *1-AMINOCYCLO-PROPANE-1-CARBOXYLATE SYNTHASE 7 (ACS7)*, downregulate ethylene synthesis, and promote root hair elongation under low levels of exogenous phosphorus [85]. The SMAX1 mutant of *Lotus japonicus* showed very short primary roots and elongated root hairs, and transcriptomic data revealed that SMAX1 downregulated the expression of *ACS7*, consistent with findings in Arabidopsis [81].

For lateral root formation, D14 and KAI2 co-regulate lateral root density which differs from other root traits in Arabidopsis [78]. In Arabidopsis, KAI2 and MAX2 have been identified as root-skewing regulators and promoters of plant growth and development through the same genetic pathway [80]. KAR signaling has been observed to regulate the synthesis of IAA and jasmonic acid (JA) positively while negatively regulating ABA synthesis, ultimately leading to the promotion of soybean root nodule formation [109]. Furthermore, the transcriptional analysis of Arabidopsis, both WT and *max2* treated with rac-GR24, has also revealed novel substances and regulatory factors affecting root hair elongation, including flavonols, HY5, MYB12, etc. [108]. The complexity of KAR signaling suggests mechanisms beyond the KAI2-dependent pathway. By enhancing root hair elongation and lateral root formation, KARs can improve nutrient uptake efficiency, particularly under nutrient-limited conditions. This regulatory function can be harnessed to develop crops with enhanced stress tolerance and improved growth performance.

### 4.4. Response to Stress

**Figure 5:**
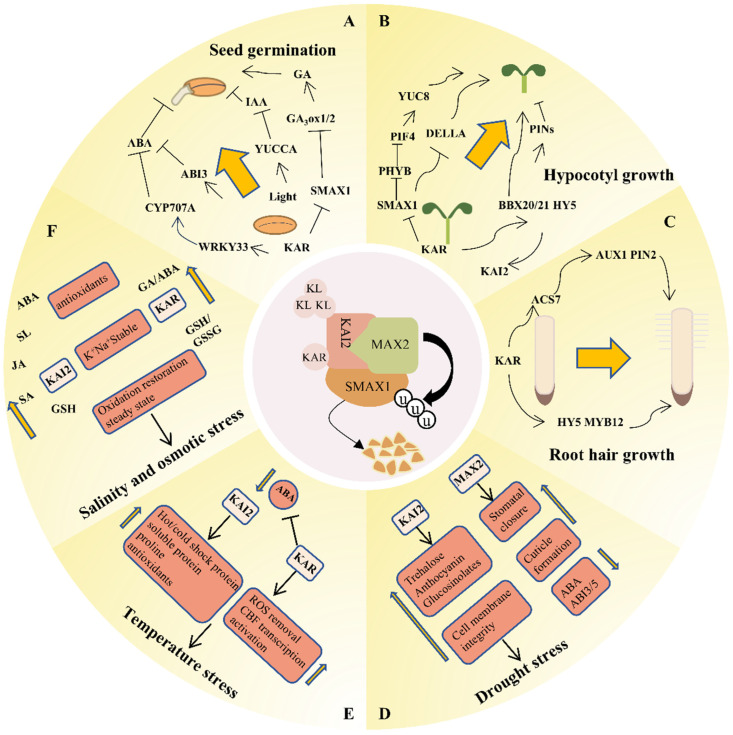
Simple working model of the main biological functions of KAR signaling. (**A**) Seed germination, (**B**) Hypocotyl growth, (**C**) Root hair growth, (**D**) Drought stress, (**E**) Temperature stress, (**F**) Salinity and osmotic stress. Arrows indicate changes in gene expression or material content, with upwards representing an increase and downwards representing a decrease. ⊥ Represents the inhibitory effect on downstream gene expression.

In addition to regulating plant growth and development, KARs also play a role in managing abiotic stresses such as drought, extreme temperatures, low light, oxidation, and other challenges [14,88]. We will now focus on three abiotic stresses regulated by the KAR signaling pathway: drought, temperature, and osmotic stress (Table 1) (Figure 5).

#### 4.4.1. Drought Stress

The role of KAR signaling in drought resistance was initially observed in the *max2* mutant [110]. The increased drought sensitivity in the *max2* mutant is due to a thinner cuticle layer and reduced response to ABA [110]. Subsequent studies on the *kai2* mutant uncovered its drought susceptibility, characterized by higher water loss, membrane damage, larger stomatal apertures, and increased cuticle permeability [111,112]. Under drought stress, the *kai2* mutant exhibited reduced anthocyanin and ABA insensitivity in stomatal closure and cotyledon opening, similar to the *max2* mutant [110,111]. Research indicated that the KAI2 pathway facilitates the biosynthesis of drought-protective compounds such as glucosinolates and trehalose [113]. Additionally, SMAX1/SMXL2 was found to negatively regulate drought resistance, as *smax1* and *smlx2* plants exhibited enhanced drought resistance and reduced leaf water loss phenotypes that can be attributed to their increased sensitivity to ABA and enhanced capacity for reactive oxygen species detoxification [86]. Collectively, these investigations corroborate the function of KAR signaling in enhancing drought resistance.

#### 4.4.2. Temperature Stress

The involvement of KAR signaling in plants’ resilience to thermal stress was initially identified in the *kai2* mutant, which showed lower germination and greater sensitivity to abiotic stress compared to WT [117]. Further, it was found that KAR signals against temperature stress by regulating heat and cold shock proteins [114,118]. Transcriptome analysis of *kai2* mutants revealed that KAI2 enhances heat resilience in Arabidopsis through the modulation of heat shock transcription factors and heat shock proteins, with transcription factors like WKRY and NAC likely involved [115]. The *kai2* mutant also exhibits a heightened rate of mortality under conditions of cold stress, which was mitigated by the overexpression of the KAI2 protein from *Sapium sebiferum* [116]. This research identified a marked elevation in the constituents of cold shock proteins, C-repeat binding factors, antioxidants, total soluble sugars, and proline in the *SsKAI2* overexpression line [116]. Silencing KAI2 and MAX1 in tomatoes using VIGS revealed that KAR primarily promotes growth and yield by interacting with ABA signaling, enhancing CBF transcriptional activation, and reducing ROS [114].

#### 4.4.3. Salinity and Osmotic Stress

Arabidopsis *kai2* seeds demonstrated reduced germination under osmotic stress, analogous to heat stress conditions, implying KAI2’s involvement in osmotic stress tolerance [117]. Studies on the *kai2* mutant under salt stress revealed phenotypic deviations, diminished biomass, heightened water loss, and escalated oxidative damage [119]. NaCl stress caused higher sodium (Na^+^) accumulation and a lower potassium (K^+^)/sodium (Na^+^) ratio in *kai2* seedlings compared to WT, indicating heightened ion toxicity [119]. Furthermore, the *kai2* mutant demonstrates reduced gene expression levels for the synthesis of salicylic and jasmonic acids, as well as the signal transduction of ABA and SLs [119]. Exogenous application of KAR_1_ can efficaciously mitigate plant stress and enhance seed germination, seedling biomass, and root growth under stressful conditions [76,117]. In the case of *Sapium sebiferum*, the implementation of KAR_1_ effectively modulated both aerobic and nutrient equilibrium while altering the expression of certain ABA signaling genes like *ABI3* and *ABI5* [120]. In wheat, the exogenous supplementation of KAR_1_ not only conserved soil nutrients through oxygen reduction but also sustained the K^+^/Na^+^ nutrient balance of seedlings [118]. The ability of KAR signaling to enhance stress tolerance has significant practical implications for agriculture, particularly in developing crops with improved resilience to environmental challenges.

### 4.5. Other Functions

KARs also play various roles in biological functions (Table 2). They contribute to disease resistance and regulate the immune response by altering the SMAX1/SMXLs composition [61]. MAX2 protein influences cytokinin and auxin levels, promoting plant callus formation and regulating light signals [60,121]. Research indicated that the growth of NaMAX2 in field conditions demonstrates autonomy from both SL and KAR signaling pathways in the regulation of high-light responses in *Nicotiana attenuata* [122]. The study revealed that the KAR pathway could promote the development and initiation of gemma cups in *Marchantia polymorpha* [123].

In Arabidopsis, rac-GR24 and SLs enhance GSNOR protein levels to promote NO signal transduction [124]. In *Salvia miltiorrhiza*, KAR_1_ induces secondary metabolite synthesis by increasing NO and JA levels [125]. The mechanism of KARs on secondary metabolite accumulation in medicinal plants remains unknown, but it has potential in agriculture [125]. KAR_1_ treatment in cabbage improves seed germination, seedling growth, and plant biomass under cadmium stress by enhancing osmotic potential and membrane stability [126]. Virus-induced gene silencing revealed that *GhSMAX1-1* and *GhSMAX1-2* suppress the growth and development of axillary buds [127].

## 5. Conclusions and Future Perspectives

Recent research has significantly enhanced our understanding of KAR signal transduction and highlighted its role in various biological functions, including plant growth, development, seed germination, and stress responses. The perception of KAR by its receptor KAI2 and the role of other proteins like MAX2 and SMXLs in the signaling pathway have been well-documented. However, several key questions remain unresolved.

First, endogenous KARs have yet to be confirmed. The synthesis and decomposition pathways, as well as the mechanisms of KAR absorption and transport in plants, are still unknown. Investigating the structure of the KAI2 receptor protein may help identify endogenous KARs [35]. Second, the downstream target genes of SMAX1/SMXL2, the core regulators of KAR signaling, remain poorly understood, particularly concerning plant stress resistance and hormone cross-talk. Recent findings suggest that SMAX1/SMXL2 regulate target genes through both transcriptional and non-transcriptional mechanisms, influencing hypocotyl development and potentially interacting with unknown proteins. Most studies focus on Arabidopsis, leaving a gap in understanding SMAX1/SMXL2 functions in other crops. Research on diverse crops could reveal structural and functional variations in these proteins. Third, the molecular mechanisms by which KAR-KL signaling confers stress tolerance and the specific targets required for this process are not fully understood. Further research is needed to elucidate complex signaling networks and downstream effectors. A practical question is whether KAR-KL signaling can be manipulated to improve plant stress tolerance. For example, low nitrogen has been shown to promote D14 phosphorylation and stabilization to inhibit rice tillering through key amino acid residues of D14-sensing SL signaling [128]. However, there is a lack of research on the specific sites of the receptor KAI2 and the key downstream regulators SMAX1/SMXL2. Given the evolutionary divergence of these proteins, species-specific studies and advances in precise gene editing could enable targeted modifications to enhance stress tolerance in crops.

In summary, KARs offer significant potential for further research. Addressing these challenges and exploring their molecular mechanisms could expand their application in agriculture, leading to the development of stress-resistant crop varieties and substantial benefits for future farming.

## Figures and Tables

**Table 1:** The role of KAR signaling in alleviating abiotic stress.

Species	Abiotic Stress	Mechanism of Tolerance	Reference
*Arabidopsis thaliana*	Drought	Reduced leaf water loss due to the increased sensitivity to ABA and enhanced detoxification ability for active oxygen	[86]
*Arabidopsis thaliana*	Drought	Cuticle thickness and ABA gene expression	[110]
*Arabidopsis thaliana*	Drought	Stomatal closure, cuticle protection, anthocyanin biosynthesis, ABA insensitivity	[111]
*Arabidopsis thaliana*	Drought	KAI2 is more efficacious in attenuating electrolyte leakage, promoting cuticle formation, and diminishing permeability compared to D14	[112]
*Arabidopsis thaliana*	Drought	Promotes the biosynthesis of glucosinolates and trehalose	[113]
*Arabidopsis thaliana*	Temperature	SMAX1 interacts with PHYB to relieve the inhibition of *PIF4*	[104]
*Solanum lycopersicum*	Temperature	Influence of ABA content	[114]
*Arabidopsis thaliana*	Temperature	Heat shock protein regulation and heat shock-dependent transcription	[115]
*Sapium sebiferum*	Temperature	Redox homeostasis, cold shock proteins	[116]
*Arabidopsis thaliana*	Osmotic	Enhanced expression of *DREB2A*, *WRKY33*, and *ERF5* genes	[117]
*Triticum aestivum*	Osmotic	Maintaining the redox homeostasis and the K^+^/Na^+^ homeostasis	[118]
*Arabidopsis thaliana*	Osmotic	Redox homeostasis, antioxidant enzyme activity	[119]
*Sapium sebiferum*	Osmotic	Regulates redox homeostasis and alters ABA signaling gene expression	[120]
*Arabidopsis thaliana*	Osmotic	D14 mediates osmotic-stress-induced SMAX1 degradation	[76]

**Table 2:** Other functions of KAR signaling.

Species	Function	Mechanism of Tolerance	Reference
*Arabidopsis thaliana*	Enhance the development of lateral roots while concurrently postponing the formation of root calli	MAX2 serves a regulatory function in mediating the interaction among cytokinin, auxin, and light signals during the onset of callus formation	[60]
*Arabidopsis thaliana*	Enhance immune defense response	Dependent on salicylic acid (SA) signaling	[62]
*Arabidopsis thaliana*	Affects wounded tissue and seed development	CK and MAX2 regulate development independently and interactively	[121]
*Nicotiana attenuata*	Adaptation to high-light response	NaMAX2 regulates the antioxidant system	[122]
*Marchantia polymorpha*	Promotes gemma cup formation and gemma initiation	Controlled by the ON/OFF switch of KAI2-related signals	[123]
*Arabidopsis thaliana*	Adjusting NO signal	rac-GR24 and SLs enhance GSNOR protein levels	[124]
*Salvia miltiorrhiza*	KAR_1_ regulates the secondary metabolism of medicinal plants	KAR_1_ induces the generation of nitric oxide (NO), jasmonic acid (JA), and T-I in the hair-like root	[125]
*Brassica oleracea*	Relieve the toxicity of cadmium	The reduction of the level of MDA, EL, and H_2_O_2_ and the improvement of the antioxidant mechanism	[126]
*Gossypium* sp.	Promote stem elongation and axillary bud development	Degradation of GhSMAX1-1 and GhSMAX1-2	[127]
